# Health Tract, No. 140

**Published:** 1865

**Authors:** 


### HEALTH TRACT, No. 140.

### SPRING-TIME.

In the early part of May, very many persons begin to feel that they are not as well as they have been. There is a degree of languor and lassitude, an indisposition to exercise, or even to read or think much, which makes life almost a drag. This ought not to be. There is no good and sufficient reason why man should not wake up to a newness of life, and embark in its business with a new energy and a new enterprise. The grass shoots up in its greenness so delightfully refreshing, that we love to look upon it; the buds swell on the trees, and the beautiful flowers unfold themselves; while the birds of the wood fill the sweet air with their rich and gladsome diapasons! And why should man alone, of all the creation, look with a languid eye upon the spring-time? It is unnatural, it is wicked, it is absurd; and it comes about in this plain matter-of-fact way. Man alive! do you see that pig yonder, lying in the corner of the fence, or at the foot of the wall, his eye half-closed, and so lazy that he can't summon up courage enough to wag his tail? An hour sooner he was not so, but was running toward the corn-crib, at the farmer's cry of "pee-gy," with the same agility that a little beggar-boy will run from you, these times, on the discovery that you have in mistake given him a dime instead of a nickel. The pig has eaten so much that he can scarcely grunt. The lassitude which comes over multitudes of humanity with the beautiful spring, is the result of eating too much. There is nothing in the spring air to cause this; for it is soft and balmy and blissful, and brings animation and a newness of life to every living thing, man only excepted!

The "modus operandi" is worthy of being studied, and well matured, by every intelligent reader. We are all kept from freezing by an internal furnace; the fuel for which is the food we eat; the living furnace, like that of our dwellings, requires more fuel in winter than in summer. Who has not, in considerable anger, abused Bridget for roasting them, by keeping up a greater fire in April than in mid-winter? and we call it perversity. But the maid does in the cellar what the mistress does in the dining-room, she simply puts the same amount of fuel in the grate or furnace daily. The maid roasts the outside of her mistress, while the mistress herself roasts her inner-man; thus she is literally between two fires. Is it any wonder that people complain of spring fever? As a remedy, Bridget opens the doors and windows and diminishes the heat, while the mistress resorts to tonics, and the master to "bitters," alias brandy-and-water, to whet up the appetite, to make the stomach call for more fuel, instead of attending to the stomach's instinct, in calling for less food. In all nature man is the biggest fool.

In spring be a strict vegetarian, be a strict cold-water man, keep clean, keep cheerful, keep out of doors, and your spring-time will not be the sleepiness of the pig, but it will be as gleeful and as gladsome as that of the sweetest birds of May.

